# Comparative analysis of research hotspots and development trends of pediatric palliative care at home and abroad based on CiteSpace: a bibliometric study

**DOI:** 10.3389/fped.2026.1688720

**Published:** 2026-03-06

**Authors:** N. I. Yuhong, L. A. N. Tianying, G. A. N. Xinyan, G. O. N. G. Daoqing

**Affiliations:** College of Public Health and Management, Guangxi University of Chinese Medicine, Nanning, Guangxi, China

**Keywords:** bibliometric study, children, CiteSpace, palliative care, visualization analysis

## Abstract

**Aim:**

To visualize and analyze the literature on palliative care for children based on Web of Science and CNKI database.

**Methods:**

The research literature on child palliative care included in the Web of Science and CNKI databases from 2000 to 2024 was searched, and the publication volume, authors, institutions, keyword clusters and emerging words of the literature were analyzed by CiteSpace 6.3.R2 software, and the publication trends, core force distribution and theme evolution paths of Chinese and foreign studies were compared.

**Results:**

A total of 1,256 articles were included, and the visual analysis structure showed that the number of publications on palliative care for children in China and abroad showed an overall upward trend, but there was a significant gap in the overall number of publications, and the number of publications in foreign countries was nearly ten times that of China. In terms of research strength, foreign countries have close cooperation with Harvard University and the Dana-Farber Cancer Institute as the core. China is concentrated in medical institutions in first-tier cities in the east, and cooperation is loose; From the analysis of keywords, it can be seen that foreign research hotspots focus on patient experience, and in recent years, they have shifted to practice optimization such as service barriers, while China focuses on methodology and service system construction.

**Conclusion:**

It is expected that the future research on global child palliative care will carry out multidisciplinary collaboration and resource integration, reduce practical barriers, improve social cognition, and artificial intelligence technology-driven and optimize needs assessment tools.

## 1 Introduction

Pediatric Palliative Care, also known as child palliative care, is defined by the International Child Palliative Care Network as a “positive and comprehensive” model of care, including physical, emotional, social, and spiritual, aiming to alleviate patient suffering, improve end-of-life quality, strengthen communication and optimize services, and support families in medical decision-making (1). In the context of contemporary global disease spectrum changes and medical technology iteration, the contradiction between the prolongation of survival and the assurance of life quality of critically ill children is intensifying. However, at present, the global child health work is still mainly oriented towards reducing mortality, and the construction of the child palliative care system is significantly insufficient.

According to surveys, more than 21 million children worldwide are affected by life-limiting diseases, and more than 8 million of them require specialized child palliative care. As a populous country, China has a particularly large population, with about 4.5 million school-age children in need of hospice care. However, there is a significant gap in the supply of children's hospice services in China, and a survey in 2017 showed that only 90,600 beneficiary children could actually be covered (2), and the existing service volume in China could only meet about 2% of the clinical needs, and the contradiction between supply and demand was sharp.

In terms of global palliative care research, European and American countries have built a relatively mature child palliative care policy system and multidisciplinary collaboration network, and academic research focuses on family decision-making models, symptom management technology innovation, and ethical dilemma. Since the promulgation of the “Guidelines for the Practice of Hospice Care in China” in 2017, the research and practice of palliative care in China is still in the exploratory stage, and the existing research is mainly aimed at adults, and there are relatively few special studies on children.

In recent years, although Chinese scholars have conducted literature analyses on the field of pediatric palliative care, most of the research data sources have been foreign literature, with a lack of direct bibliometric comparative analysis between Chinese and foreign literature on pediatric palliative care. This leaves a crucial research gap to be addressed: what exactly are the differences in research agendas, evolutionary paths, and cutting-edge areas of pediatric palliative care between China and other countries? And how have their respective development trajectories and frontier trends evolved? To systematically answer these questions, this study conducts data mining and bibliometric analysis using the CiteSpace visualization software, based on 1,256 Chinese and English literatures published from 2000 to 2024 retrieved from the Web of Science and China National Knowledge Infrastructure (CNKI) databases. The uniqueness and purpose of this study lie in, by means of information visualization, an in-depth comparison of the research hotspots, development trends, and core differences in the field of pediatric palliative care between China and foreign countries over the past three decades. It thereby provides key insights for a comprehensive understanding of the research status in this field and offers references for the future research directions of pediatric palliative care in China.

## 2 Data sources and methods

### 2.1 Data sources

In 2002, the World Health Organization (WHO) released an important update to the definition of palliative care, explicitly stating that its principles could be applied in the early stages of diseases. This landmark shift greatly expanded the scope of palliative care and significantly boosted academic attention and research growth for pediatric palliative care as an independent field. The year 2000 was chosen as the starting point of the study to fully capture the evolution of global research since this critical period. The endpoint was set at 2024 to incorporate the latest academic achievements, thereby ensuring the timeliness of the analysis on current research hotspots and future trends.

**Figure 1:**
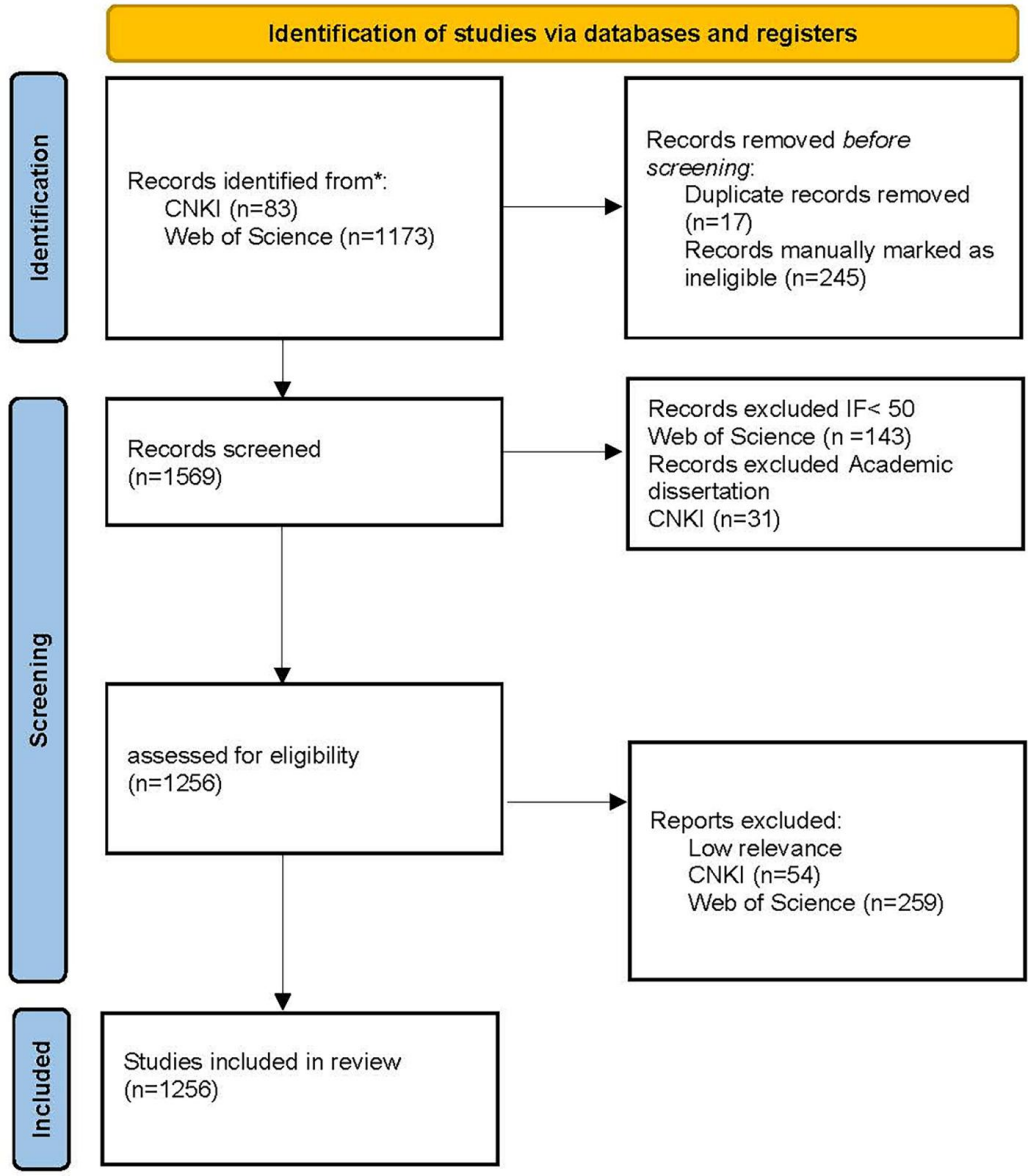
Preferred reporting items for systematic reviews and meta-analyses flow diagram.

From Figure 1, the literature data in this paper are obtained from Web of Science and CNKI databases. In Web of Science, the search format was set to TS = (“Pediatric Palliative Care” OR “Pediatric Hospice Care”), the document type was selected as “Article”, the search period was from January 1, 2000 to December 31, 2024, and 1,173 articles were obtained after sorting out irrelevant and duplicate data. The Chinese literature published from January 1, 2000 to December 31, 2024 was searched in the CNKI database with the themes “children's hospice care”, “children's hospice care” and “child palliative care”, and the academic journals were screened as SCI source journals, EI source journals, Peking University core journals, CSSCI source journals and CSCD source journals.

### 2.2 Research methods

To systematically depict and compare the research landscape of pediatric palliative care at home and abroad, this study adopts the method of bibliometric analysis. As a quantitative analytical approach, bibliometrics takes various external characteristics of scientific and technological literature as its research object. It employs mathematical and statistical methods to describe, evaluate and predict the current status and development trends of science and technology. Its primary feature lies in the fact that the output must be quantitative information content (3), and its core objective is not to conduct an in-depth interpretation or synthesis of specific conclusions from a small number of original studies, but to macroscopically reveal the overall structure, evolutionary trajectory, research hotspots and future frontiers of a knowledge domain explicitly stating that its principles could be applied in the early stages of diseases. This landmark shift greatly expanded the scope of palliative care and significantly boosted academic attention and research growth for pediatric palliative care as an independent field. The year 2000 was chosen as the starting point of the study to fully capture the evolution of global research since this critical period. The endpoint was set at 2024 to incorporate the latest academic achievements, thereby ensuring the timeliness of the analysis on current research hotspots and future trends.

The analysis report function of Web of Science and CNKI database was used to count the annual publication volume, and the obtained literature records and citations were exported in plain text format, and author analysis, institutional collaboration analysis, keyword clustering analysis and emergence analysis were carried out using CiteSpace 6.3.R2 software. In the process of analysis, the measurement parameters such as citation frequency, mediation centrality and emergence intensity are set, and visual maps such as the number of publications, authors, institutions, keyword clustering and emergence words are drawn according to the parameters, and then the research hotspots and development trends in the field of palliative care for children in China and abroad are compared and analyzed.

## 3 Results analysis

### 3.1 Annual publication volume and country analysis

The number of Chinese and English papers published can intuitively show the development trend, activity and differences between Chinese and foreign children's palliative care research in terms of time and geography. As can be seen from Table 1, the top five countries with the largest number of English literature publications are the United States (732 articles), Canada (147 articles), Germany (61 articles), the United Kingdom (54 articles) and Australia (41 articles), of which the United States ranks first with 732 papers, indicating that the United States is significantly ahead in research investment and academic output in the field of child palliative care research. In addition, although the number of articles published in the UK is only 54, its mediation centrality is 0.37, indicating that the UK is in a key position in the global child palliative care research network and an important bridge for cross-border cooperation and knowledge dissemination.

**Table 1:** Top five countries in the web of science database for the number of publications in the field of child palliative care research.

Rank	Country of Publication	Betweenness Centrality	Publication Volume (articles)
1	United States	0.18	732
2	Canada	0.26	147
3	Germany	0.06	61
4	United Kingdom	0.37	54
5	Australia	0.28	41

**Figure 2:**
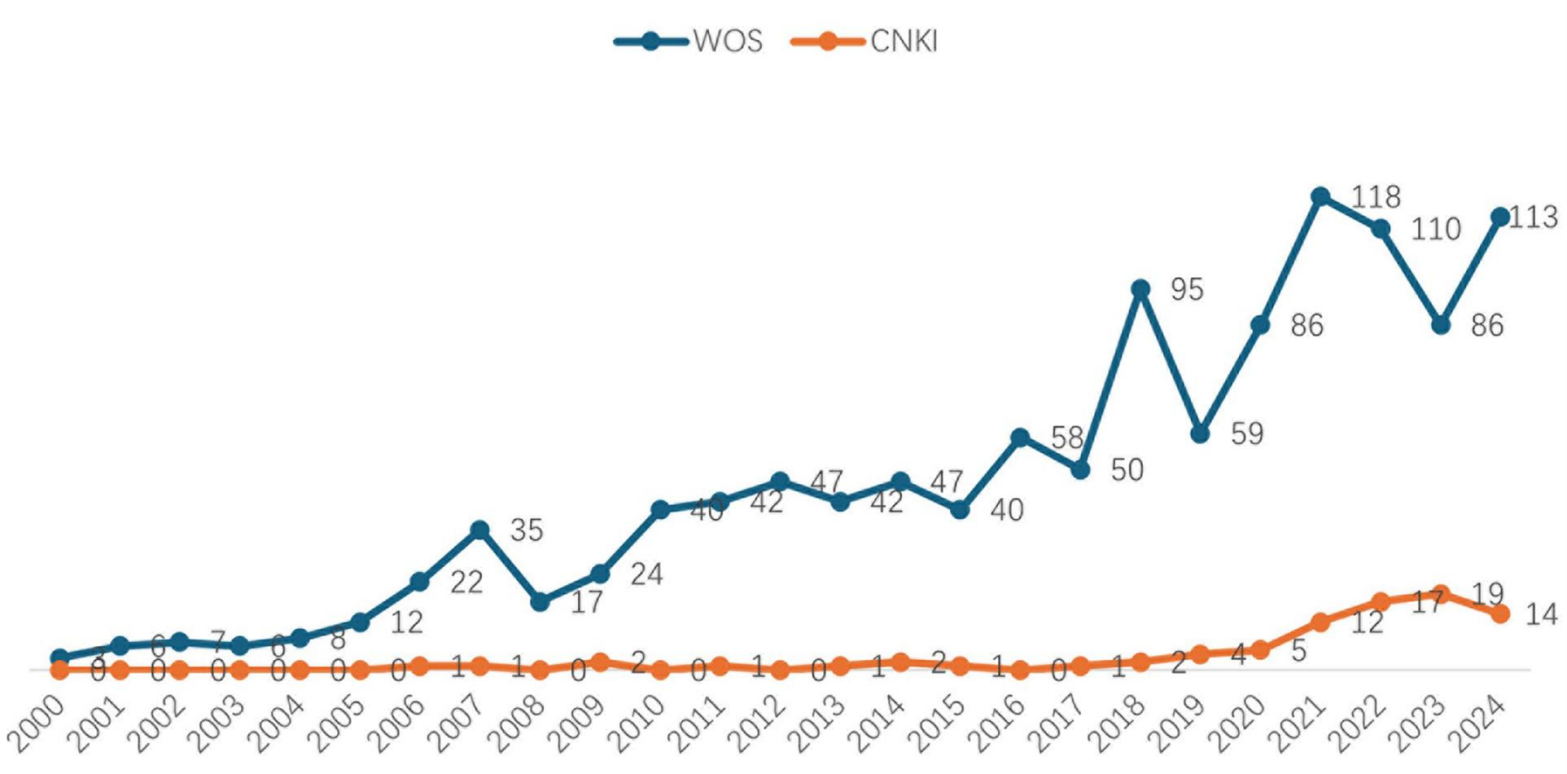
The number of research literature on children's palliative care at home and abroad is trending.

As can be seen from Figure 2, the number of English literature published has gradually increased since 2000, with a small peak in 2008 and 2018, and a peak of 118 articles in 2021. The number of publications in Chinese literature has been at a low level before 2010, and since 2010, the number of publications has begun to grow slowly, indicating that China's research in the field of children's palliative care started late, but in recent years, the attention has increased, and the number of studies has gradually increased, but the number of publications is still relatively small compared with foreign countries. In general, global research on palliative care for children is constantly evolving, but foreign research started earlier, more mature and fluctuated, and Chinese research started late but is gradually catching up, and there is still a lot of room for development in the future.

### 3.2 Author analysis

**Figure 3:**
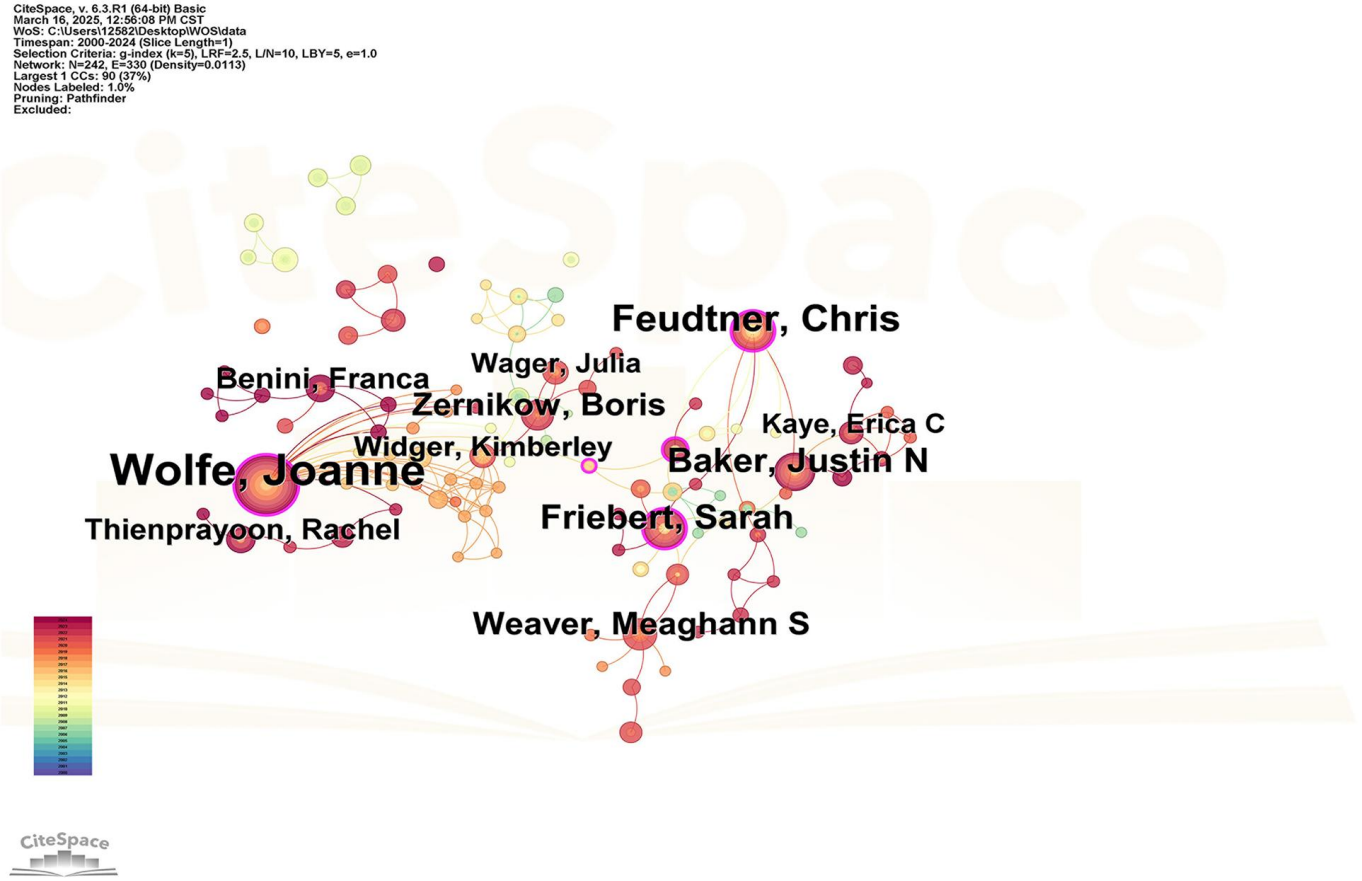
Visualization of the authors of the child hospice study in the web of science database.

**Figure 4:**
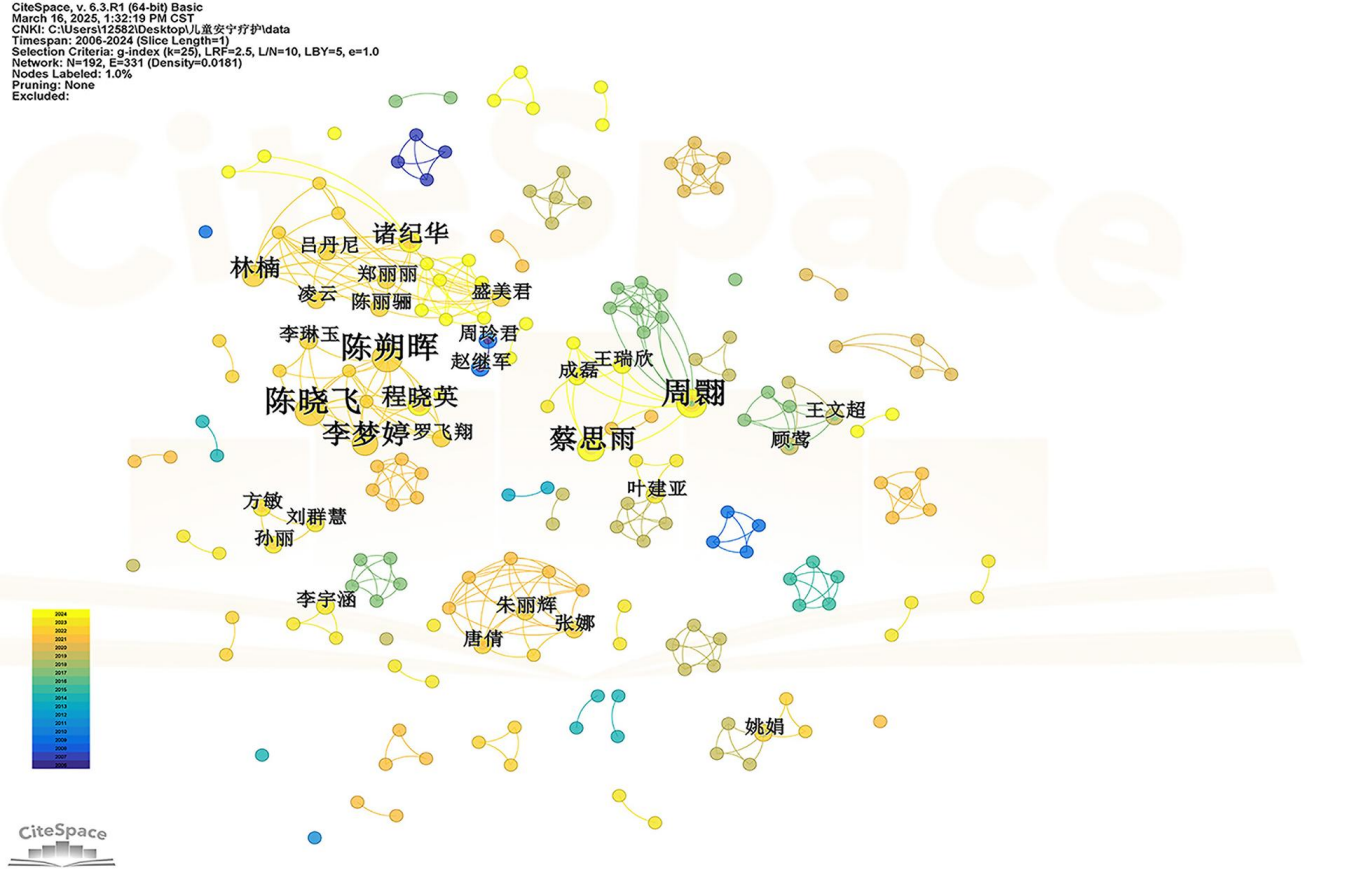
Visualization of the authors of the child hospice study in the CNKI database.

Through the analysis of author collaboration, it is helpful to identify the leading figures in the field of child palliative care, understand the cooperation mode and communication between authors, and provide clues for finding potential cooperation partners and learning from excellent research experience. In the Web of Science database, a total of 242 authors have published literature related to the field of child palliative care research in the past 30 years, resulting in a total of 330 collaborative connections, and the top five authors in terms of publication volume are Wolfe Joanne (76 articles), Feudtner Chris (35 articles), Baker Justin N (25 articles), Friebert Sarah (25 articles), and Weaver Meaghann S (20 articles). In addition, it can be seen from Figure 3 that Wolfe Joanne is in an absolute central position and has close ties with other authors, indicating that she is at a high level of influence in the field of foreign children's palliative care research, and the content and methodological ideas of the research are worthy of reference and attention. In the CNKI database, a total of 192 authors have published literature related to the field of child palliative care research in the past 30 years, and a total of 331 cooperative links have been generated, and the top five authors in terms of number of publications are Chen Shuohui (5 articles), Zhou Huan (5 articles), Chen Xiaofei (5 articles), Cai Siyu (4 articles), and Li Mengting (4 articles). Figure 4 shows that Chen Zhaohui, Li Miaojuan and other authors are in a relatively core position, indicating that their teams play a key role in the field of children's hospice care research in China. In general, the foreign author cooperation network presents a close structure centered on a small number of core authors, while the Chinese author cooperation network is relatively scattered, although there are some core authors, but as a whole, there is no close cooperation network like foreign countries with a few core authors as the absolute center, and cross-team cooperation is insufficient.

### 3.3 Institutional analysis

**Figure 5:**
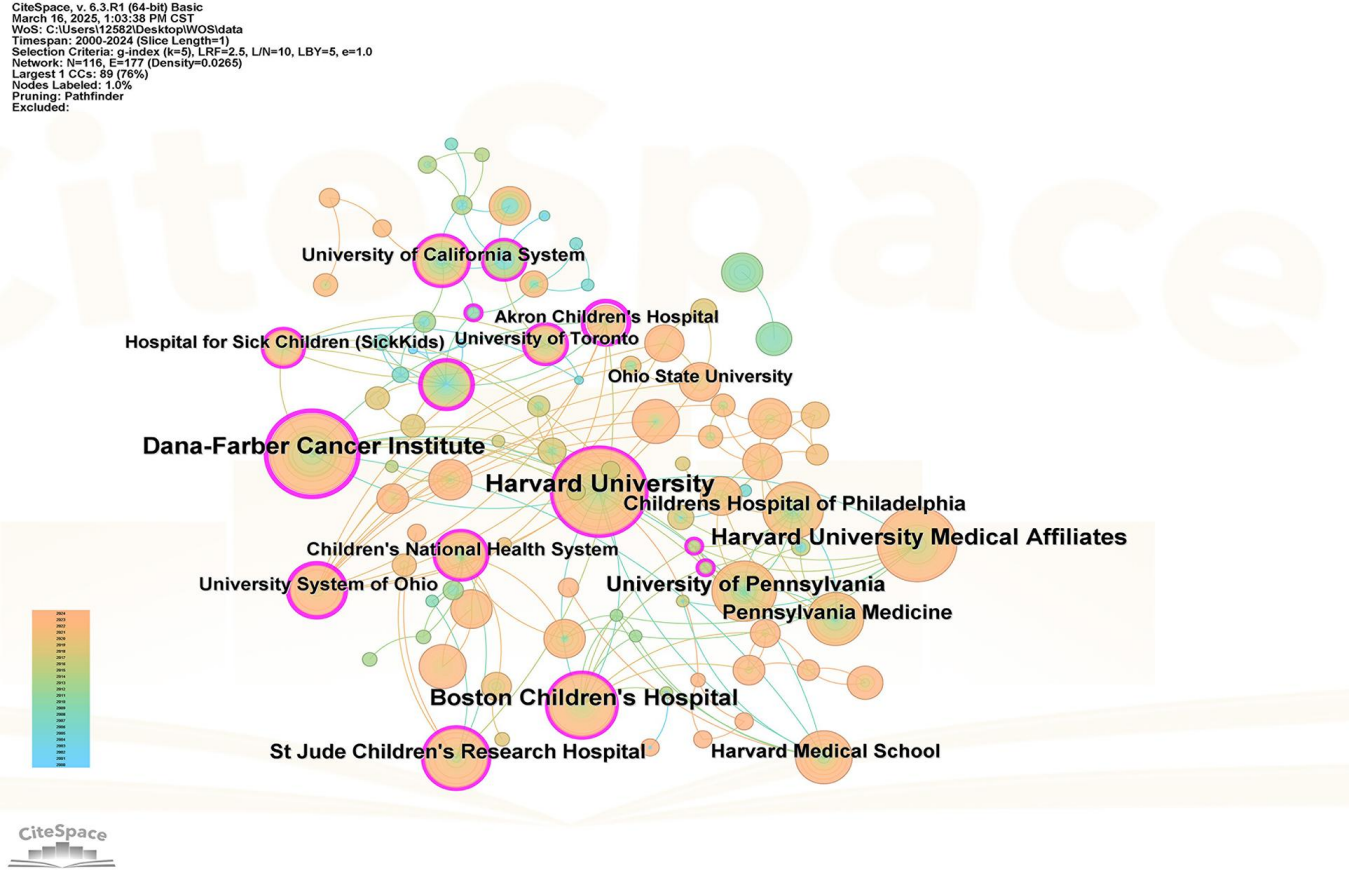
Visualization of the child hospice research institution in the web of science database.

**Figure 6:**
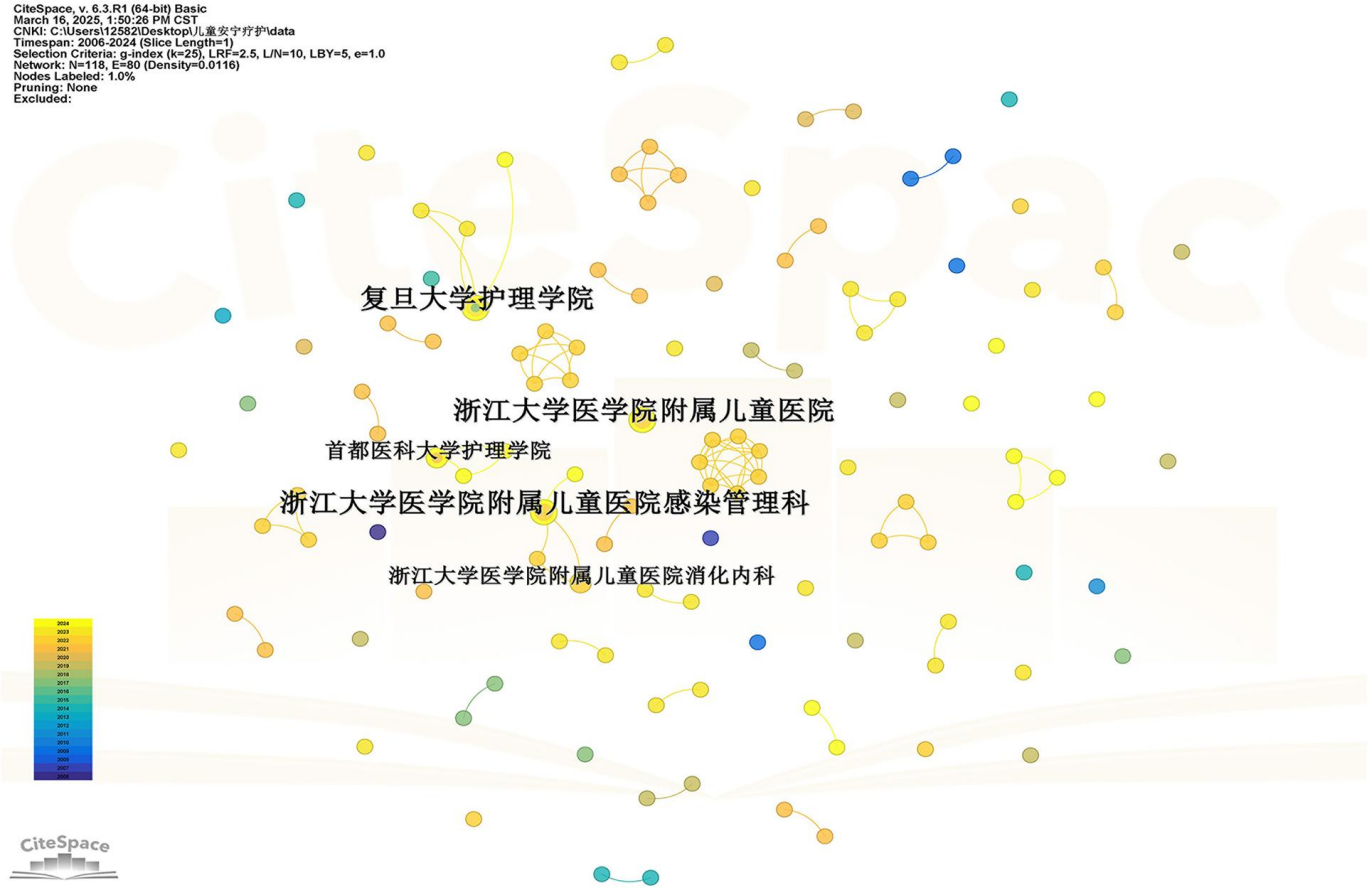
Visualization map of child hospice research institutions in the CNKI database.

Through the analysis of institutional cooperation, the distribution of the main research forces in this field and the degree of cooperation can be revealed, which provides a reference for cooperation and exchange between institutions and resource integration. In the figure, one node represents one issuing institution, the connection between nodes represents the cooperation between the issuing institutions, and the thickness of the connection represents the close cooperation between the issuing institutions. According to the English database research institution cooperation map, a total of 118 research institutions were involved, with a total of 177 cooperation connections, of which Harvard University (Harvard University) published the most articles (139 articles), followed by the Dana-Farber Cancer Institute (113 articles). As can be seen from Figure 5, there are extensive connections between many foreign institutions, and the connections between some nodes are thicker, indicating that there is a relatively common and close cooperative relationship between foreign institutions in the research of child hospice care. The cooperation map of Chinese database research institutions shows that a total of 118 research institutions are involved, and a total of 80 cooperation links have been generated, among which the Children's Hospital Affiliated to Zhejiang University School of Medicine, the School of Nursing of Fudan University and Capital Medical University have the largest number of articles. As can be seen from Figure 6, there are a certain number of connections among Chinese institutions, but the number is relatively small and the links between nodes are thin. This indicates that the scope of cooperation among relevant Chinese institutions is relatively narrow, and the depth of cooperation needs to be further strengthened.

### 3.4 Keyword analysis

#### 3.4.1 Keyword clustering analysis

Keyword clustering analysis is a crucial method for exploring major research trends and hotspots in a specific discipline. In CiteSpace visualization analysis software, the evaluation of clustering effectiveness relies on two parameters: modularity value(Q-value) and average silhouette coefficient (S-value). When the Q-value is greater than 0.3, it indicates a significant cluster structure; an S-value above 0.5 means the clustering is reasonable (4). In this study, the Q-values for the Web of Science and CNKI database keyword clustering maps were 0.7531 and 0.7594, respectively, and the S-values were 0.9086 and 0.9063, respectively. Therefore, the clustering of the maps is generally reasonable, and their cluster structures are significant. Each cluster contains several closely related terms, and a smaller cluster number indicates that the cluster contains more keywords. Within each cluster, one keyword is assigned the highest value and is then selected as the representative label for that cluster.

**Figure 7:**
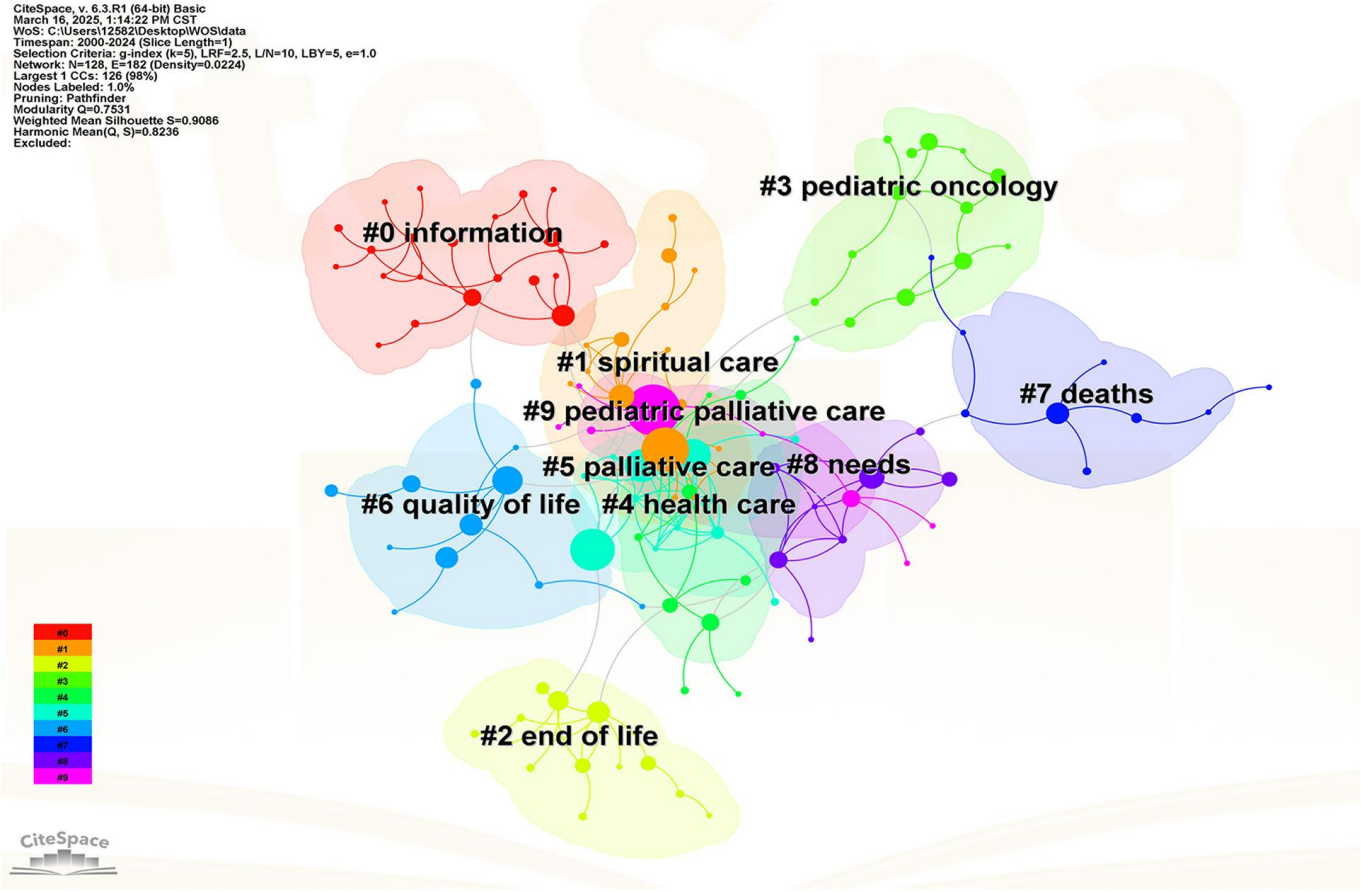
Visualization map of keyword clustering of child hospice research in the web of Science database.

Figure 7 shows that in the Web of Science database, after removing the search terms “Pediatric Palliative Care” and “Pediatric Hospice Care”, the keyword clusters of child palliative care research literature are mainly “information”, “spiritual care”, “pediatric oncology”, “quality of life”, “ end of life”, “deaths”, “needs”, “health care”. The keyword clustering results show that the core of foreign children's palliative care research focuses on patient experience and needs, psychosocial support and overall care at the end of life, and emphasizes the combination of medical care and humanities. At the same time, it also shows the characteristics of multidisciplinary collaboration, covering medicine, psychology, sociology and other fields. Finally, the direct mention of “deaths” and “end of life” also reflects the openness of foreign studies to end-of-life issues.

**Figure 8:**
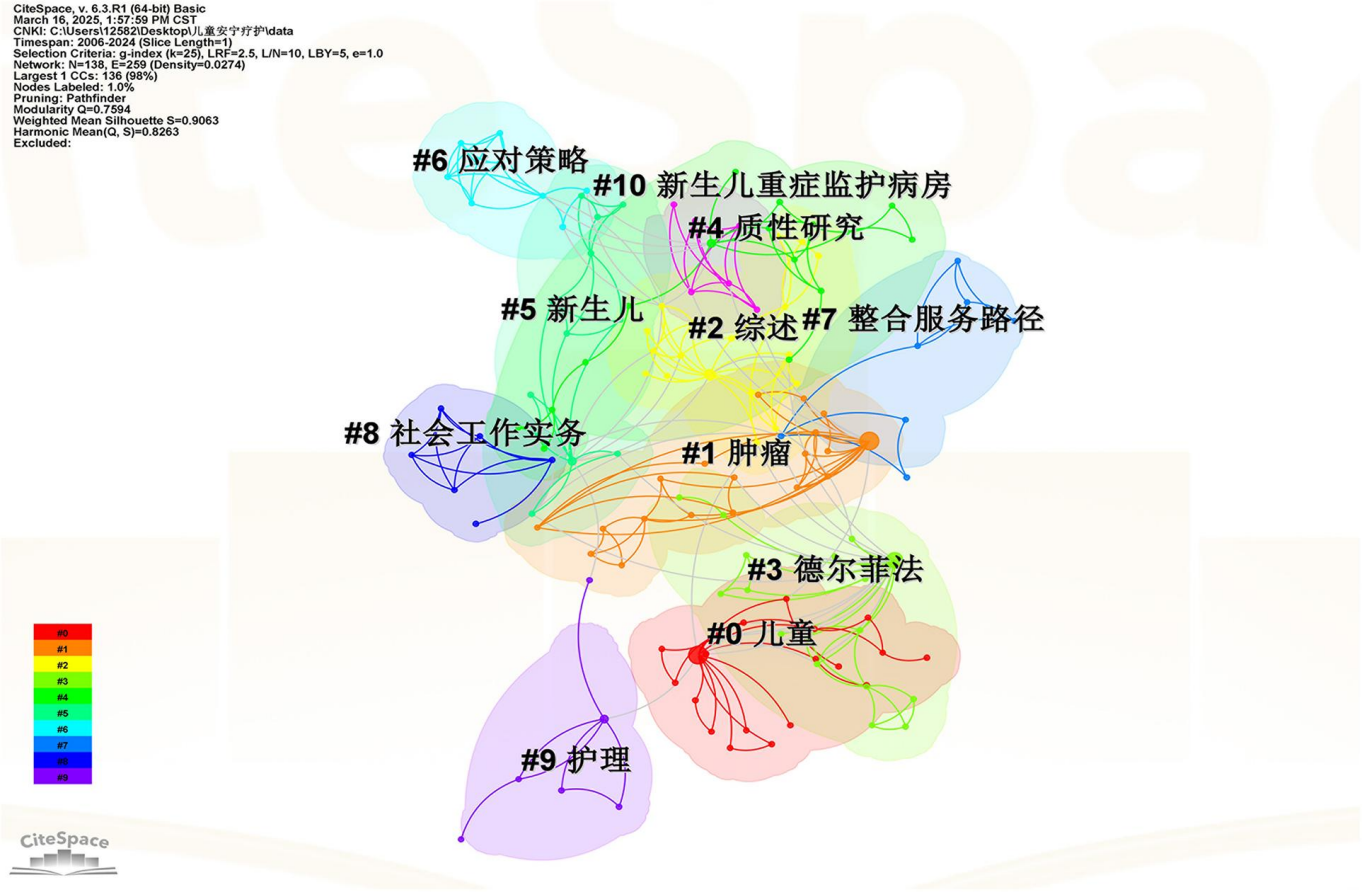
In the CNKI database, the keyword clustering visualization map of child hospice care.

As shown in Figure 8, after excluding the search term “child palliative care”, the keyword clusters of child palliative care research literature were mainly “tumor”, “review”, “Delphi method”, “qualitative research”, “neonate”, “coping strategies”, “integrated service paths”, “social work practice”, “nursing”, and “neonatal intensive care unit”. The keyword clustering results show that the research in the field of children's palliative care in China focuses on methodology and service construction. Secondly, research is more focused on specific diseases or special groups, focusing on practical exploration in subdivided fields. Finally, Chinese research also emphasizes the integration of service paths, emphasizing cross-departmental collaboration and optimizing service processes.

In general, there are great differences between research in the field of palliative care for children in China and abroad. In terms of development, foreign research has entered a mature stage of deepening individual care and multidisciplinary collaboration, while China still focuses on methodological exploration and service framework construction. In terms of research centers, foreign countries are patient-centered, and China is centered on the construction of service systems.

#### 3.4.2 Keyword emergence analysis

**Figure 9:**
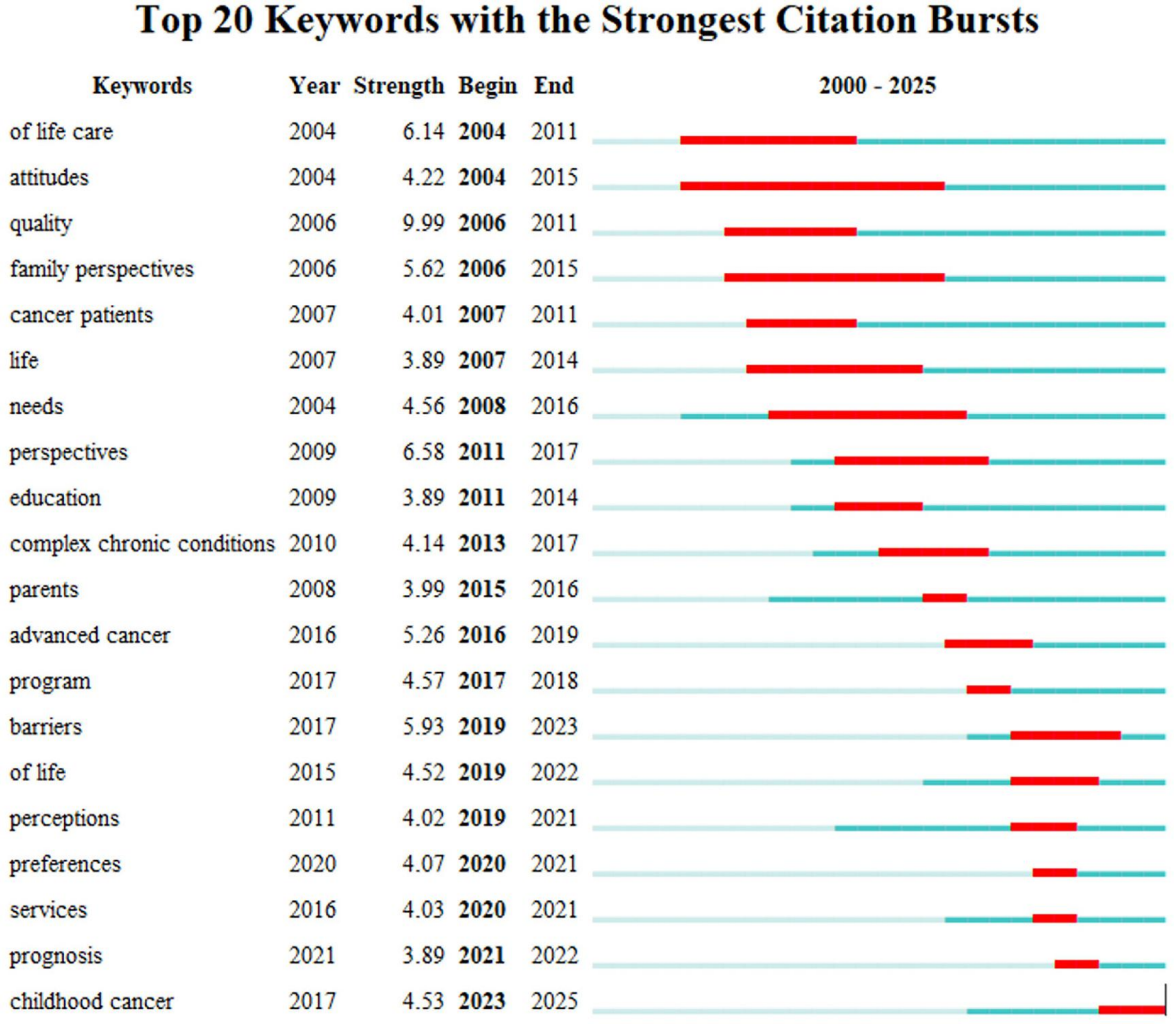
Keyword emergence analysis of child hospice care research in the Web of science database.

The emergence analysis of keywords can be used to identify emerging concepts and research topics in a certain academic field, so as to reveal the research focus or emerging research trends in the period, which is of great significance for exploring hot topics and predicting cutting-edge trends in the field of child palliative care. As can be seen from Figure 9, the keywords “quality” (strength 10.24) and “family perspectives” (strength 5.55) in the Web of Science database ran from 2006 to 2015, indicating that foreign studies have long paid attention to patients' quality of life and family participation. In recent years, the emergence of the words “barriers” (strength 5.61, 2019–2024), “perceptions” (2019–2021), and “preferences” (2020–2021) shows that the focus of research has shifted to barriers, cognitive differences and personalized needs of patients and families in service implementation, reflecting refined exploration at the practical level.

**Figure 10:**
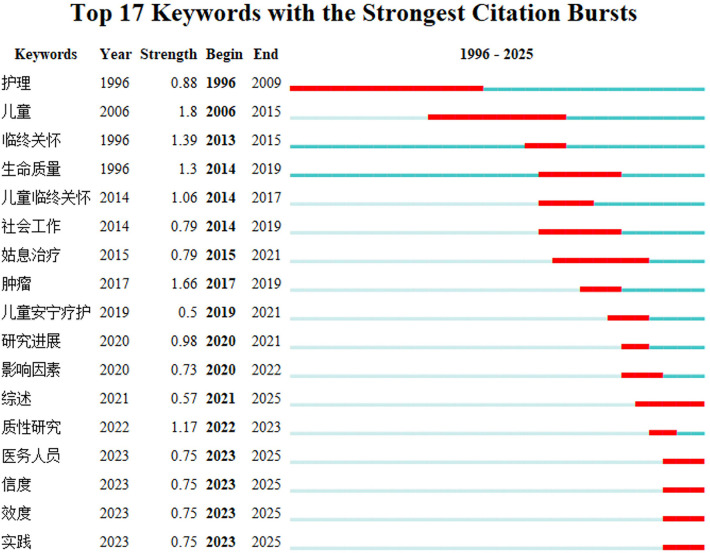
Keyword emergence analysis of children's hospice care research in CNKI database.

Figure 10 shows that in the CNKI database, the early emergence words “hospice care” (2006–2019) and “children” (2006–2015) indicate that China initially introduced international concepts, and then gradually refined to localized topics such as “children's palliative care” (2019–2021). In recent years, the high-frequency emergence words “qualitative research” (2022–2024), “research progress” (2020–2021), and “influencing factors” (2020–2022) reflect China's emphasis on the standardization of research methodologies and service frameworks, emphasizing scientific and systematic nature.

In general, the research on palliative care in China and abroad presents an evolutionary path of “deepening of concept and optimization of practice”, with foreign research focusing on life care concepts, social attitudes and service quality in the early stage, and focusing on exploring service barriers in recent years, reflecting systematic research from theory to clinic. In recent years, the terms “qualitative research” and “influencing factors” have emerged, indicating the importance of scientific research methods and localized practice evaluation.

## 4 Discuss

With the advancement of medical technology and the growth of social demand, children's palliative care as an important field to improve the quality of life of critically ill children, its research and practice need to be deepened and innovated urgently.

### 4.1 Multidisciplinary collaboration and resource integration

Multidisciplinary collaboration is of great significance in the development of palliative care. Palliative care was carried out earlier in foreign countries, and the multidisciplinary teamwork model with medical, nursing, psychological and social work as the core has matured. The 2017 National Comprehensive Cancer Network (NCCN) Clinical Practice Guidelines for Palliative Care emphasize that multidisciplinary teams should provide physical, mental, spiritual, and social high-quality hospice care services to patients and their families in a specific survival period (5). Hill DL et al. successfully developed a comprehensive interdisciplinary team intervention through collaborative design that improves the initiation of palliative care for pediatric oncology patients while promoting collaboration between different disciplines (6). Future foreign research will further deepen interdisciplinary collaboration, especially in the areas of symptom management, family decision support, and ethical dilemma resolution in children with cancer. In China, efforts need to be made to promote the construction of regional cooperation networks, integrate resources from hospitals, communities, universities and social charitable organizations, and establish a collaborative “hospital-community-home” service network, so as to address the current problems of loose inter-institutional cooperation and uneven resource allocation. In addition, it is necessary to introduce advanced foreign experience and, in combination with the characteristics of Chinese local culture, build a family-centered decision support system and an interdisciplinary team cooperation model. These will serve as important directions for future research on pediatric palliative care in China.

### 4.2 Reduce barriers to practice and enhance social awareness

At present, the promotion of children's hospice care services faces multiple practical obstacles. Foreign scholar Haines ER systematically summarizes the barriers to pediatric palliative care into four dimensions: lack of consistent and sufficient funding mechanisms at the policy/payment level, lack of pediatric palliative care programs and labor at the health system level, difficulty in integrating palliative care into the existing pediatric oncology care model at the organizational level, and lack of pediatric palliative care knowledge at the social cognitive level (7). In China, the practice of palliative care for children also faces severe challenges. Taking Shanghai as an example, Chinese scholar Zhang Zhouyixin conducted a survey of 469 pediatric nurses, and the survey revealed that the overall knowledge of Chinese medical staff on children's hospice care is not high (2),. Based on the challenges of current research and practice, Future research should explore the feasibility and operational mechanisms of establishing a National Collaborative Network for Pediatric Palliative Care Research. Meanwhile, it should focus on building a hierarchical standardized training system, developing palliative care education courses tailored to different groups such as college students, practicing physicians and nurses, and adopting innovative training methods including problem-based learning and virtual reality simulation to establish an efficient professional education model for palliative care. At the socio-cultural level, on the one hand, qualitative research methods should be employed to explore the cultural roots that hinder the acceptance of palliative care services. On the other hand, it is necessary to design and evaluate the actual effectiveness of health communication strategies such as popular science videos and life education picture books, so as to identify effective paths for reshaping public perception of palliative care and enhancing social acceptance.

### 4.3 AI-powered and optimized needs assessment tools

Artificial intelligence technology has been integrated into the field of palliative care for children. The impact of AI-based smartphone apps on cancer pain management by foreign scholars such as Kamdar demonstrates the potential benefits of AI technology in optimizing pain management (8). Chinese scholar He Longtao has built an AI + hospice care social work online service WeChat mini program to meet the diversified needs of Chinese patients and their families (9). As an important branch of palliative care, using artificial intelligence technology to optimize symptom management and remote home care is also one of the key directions for the optimization and research of global children's hospice care services in the future.

At present, there are widely used and mature pediatric palliative care screening tools in foreign countries, such as the pediatric palliative care screening scale developed by Bergstraesser et al. (10), and the pediatric complex clinical assistance needs assessment form developed by Lazzarin et al (11). Although some Chinese scholars have actively explored the screening tools for children's hospice care, such as Wang Caoyuan et al. compiled a quality evaluation scale for hospice care in end-stage NICU (12), however, whether in China or abroad, the existing children's hospice screening tools focus more on the needs of child caregivers and the evaluation of the quality of children's hospice care services, ignoring the evaluation of the child's “self” needs. Future research urgently needs to develop screening tools that can directly assess children's own needs (such as psychological support, pain expression, etc.), and truly realize child-centered child palliative care services.

## 5 Conclusion

This study uses CiteSpace to analyze 1,256 articles related to child palliative care in the Web of Science and CNKI databases from 2000 to 2024, and conducts an in-depth analysis of the annual publication volume, author and institutional cooperation network, clustering keywords and emerging keywords in this field, and obtains the following conclusions: (1) From the perspective of the number of publications, the overall number of publications in the global research on child palliative care is on the rise. However, the overall number of publications shows a significant gap, compared with China, the number of foreign publications exceeds that of China by nearly ten times, and the research started earlier and the development is more mature. China's research on children's palliative care started late, with less than 100 articles, but in recent years, the attention has increased, and the number of studies has gradually increased, and there is a lot of room for future development. (2) From the perspective of research strength, the research on palliative care for children in foreign countries is mainly distributed in developed countries such as the United States, Canada and Western Europe, with Harvard University and the Dana-Farber Cancer Institute as the core, forming a close cooperation network; The research on children's palliative care in China is mainly distributed in Zhejiang, Shanghai, Beijing and other eastern first-tier cities, with the Children's Hospital Affiliated to Zhejiang University School of Medicine, the School of Nursing of Fudan University and the School of Nursing of Capital Medical University as the core. The top contributors to the global field of child palliative care research are Wolfe Joanne, Feudtner Chris, Friebert Sarah, Chen Shuohui, Zhou Huan and Chen Xiaofei. (3) From the perspective of research hotspots, the research on palliative care for children in foreign countries focuses more on patient experience and has a wide range of disciplines. The research on children's palliative care in China focuses on methodology and service system construction, and the service scope focuses on specific groups and scenarios. It is expected that in the future, global research will be carried out on multidisciplinary collaboration and resource integration, reducing practical barriers and improving social cognition, and artificial intelligence technology driving and optimizing needs assessment tools.

## Data Availability

The datasets presented in this study can be found in online repositories. The names of the repository/repositories and accession number(s) can be found in the article/Supplementary Material.
