# Correction: Comparative analysis of research hotspots and development trends of pediatric palliative care at home and abroad based on CiteSpace: a bibliometric study

**DOI:** 10.3389/fped.2026.1826546

**Published:** 2026-03-23

**Authors:** N. I. Yuhong, G. A. N. Xinyan, L. A. N. Tianying, G. O. N. G. Daoqing

**Affiliations:** College of Public Health and Management, Guangxi University of Chinese Medicine, Nanning, Guangxi, China

**Keywords:** bibliometric study, children, CiteSpace, palliative care, visualization analysis

The funder “Research Project of Guangxi Zhuang Autonomous Region Bureau of Disease Control and Prevention - Construction and Application of Guangxi Hepatitis B Prevention and Control Information Platform Based on Artificial Intelligence (0502405501)” was erroneously omitted.

The original version of this article has been updated.

