# Supplementary figures and images for: Comparative analysis of research hotspots and development trends of pediatric palliative care at home and abroad based on CiteSpace: a bibliometric study
Source: Front Pediatr. 2026 Mar 6;14:1688720. doi: 10.3389/fped.2026.1688720 (PMC13002801; doi:10.3389/fped.2026.1688720)

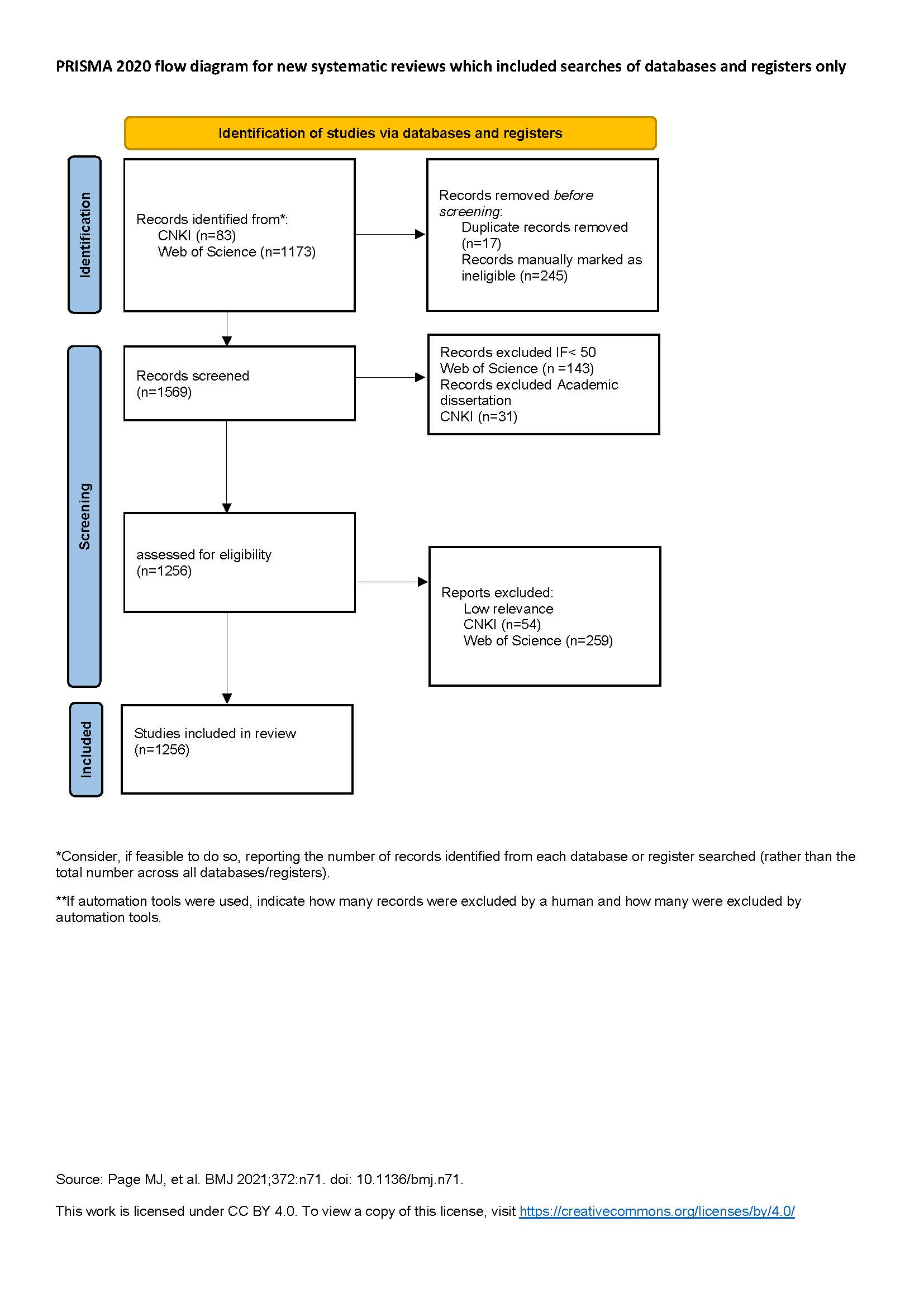

Supplement: Supplementary file 1 [file Image1.tif]
